# Supplementary material for: The heart is a resident tissue for hematopoietic stem and progenitor cells in zebrafish
Source: Nat Commun. 2024 Aug 31;15:7589. doi: 10.1038/s41467-024-51920-7 (PMC11366026; doi:10.1038/s41467-024-51920-7)
Supplement: Supplementary file 1 — Supplementary Information [file 41467_2024_51920_MOESM1_ESM.pdf]

The heart is a resident tissue for hematopoietic stem and progenitor cells in zebrafish

Dorothee Bornhorst, Amulya V. Hejjaji, Lena Steuter, Nicole M. Woodhead, Paul Maier, Alessandra Gentile, Alice Alhajkadour, Octavia Santis Larrain, Michael Weber, Khrievono Kikhi, Stefan Guenther, Jan Huisken, Owen J. Tamplin, Didier Y. R. Stainier,

Felix Gunawan

Table of contents

Supplemental Figure 1 2

Supplemental Figure 2 4

Supplemental Figure 3 5

Supplemental Figure 4 6

Supplemental Figure 5 7

Supplemental Figure 6 9

Supplemental Figure 7 10

Supplemental Figure 8 12

Supplemental Figure 9 13

Supplemental Figure 10 14

Supplemental Figure 11 15

Supplemental Figure 12 17

Supplemental Table 1 18

Supplemental Table 2 20

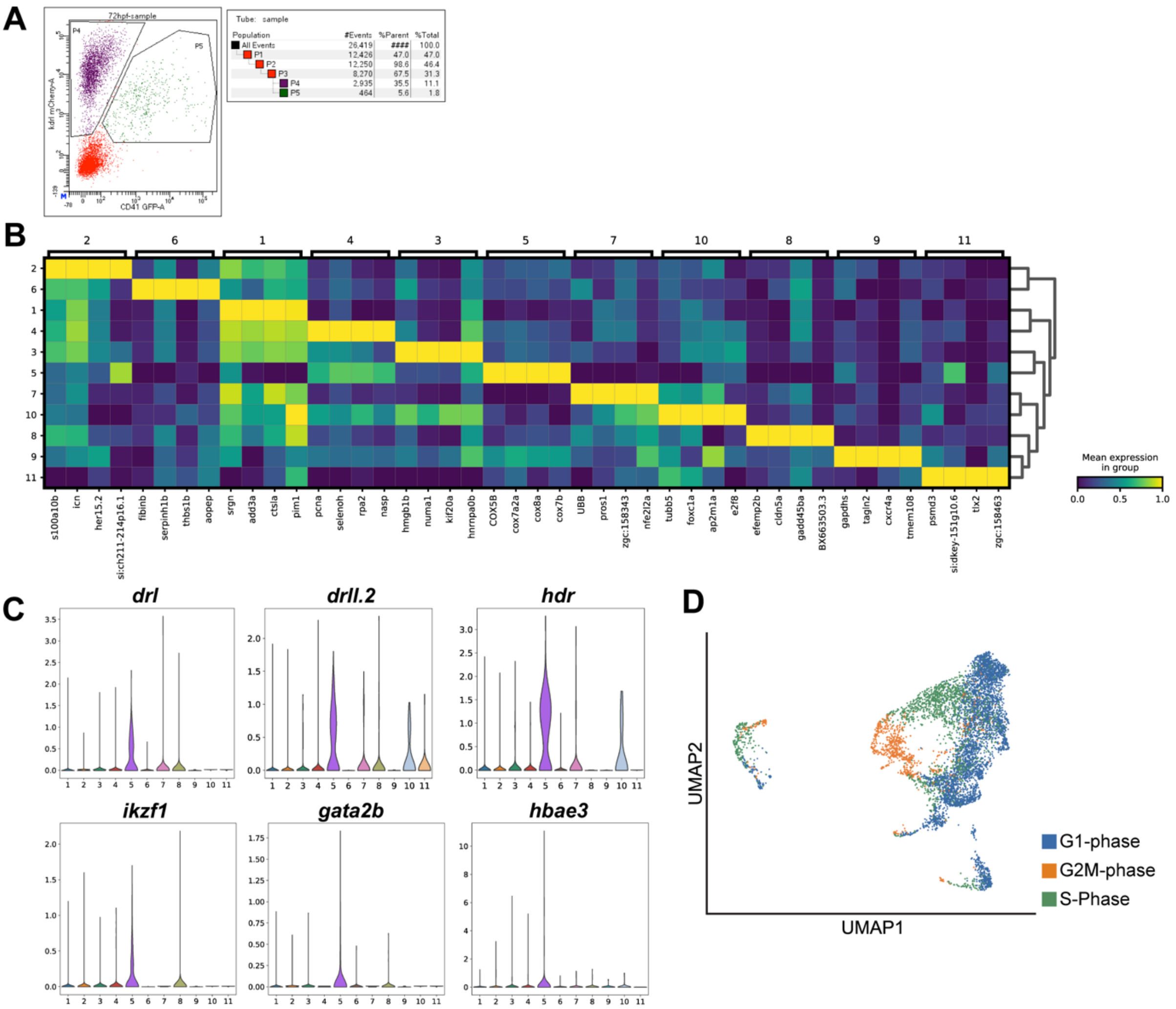
A
72hpf-sample
P4
P5
kdrl mCherry-A
CD41 GFP-A
Tube: sample
Population #Events %Parent %Total
All Events 26,419 #### 100.0
P1 12,426 47.0 47.0
P2 12,250 98.6 46.4
P3 8,270 67.5 31.3
P4 2,935 35.5 11.1
P5 464 5.6 1.8
B
2 6 1 4 3 5 7 10 8 9 11
s100a10b icn her15.2 si:ch211-214p16.1 fibinb serpinh1b thbs1b aopep srgn add3a ctsla pim1 pcna selenoh rpa2 nasp hmgb1b numa1 kif20a hnrnpa0b COX5B cox7a2a cox8a cox7b UBB pros1 zgc:158343 nfe2l2a tubb5 foxc1a ap2m1a e2f8 efemp2b cldn5a gadd45ba BX663503.3 gapdhs tagln2 cxcr4a tmem108 psmd3 si:dkey-151g10.6 tlx2 zgc:158463
Mean expression in group
0.0 0.5 1.0
C
drl
drll.2
hdr
ikzf1
gata2b
hbae3
D
UMAP2
UMAP1
G1-phase
G2M-phase
S-Phase

Supplemental Figure 1. Single-cell RNA sequencing analysis reveals diverse endocardial cell clusters. (A) Representative flow cytometry gating strategy to prepare endocardial samples from extracted hearts. Dissociated hearts of *Tg(cd41:GFP), Tg(kdrl:nls-mCherry)* zebrafish were isolated and the gating strategy includes isolation of single cells identified via FSC-A and FSC-H and then by SSC-W and SSC-H. Two gates are separated with cells expressing only *Tg(kdrl:*nls-mCherry) or both *Tg(kdrl:*nls-mCherry) and *Tg(cd41*:GFP). 35.5% and 5.6% of live cells extracted from the hearts showed mCherry expression alone or both GFP and mCherry, respectively. Both samples were combined and immediately processed for the single-cell RNA sequencing run. FSC-A, Forward Scatter Area; FSC-H, Forward Scatter Height; SSC-A, Side Scatter Area; SSC-H, Side Scatter Height; Side SSC-W, Side Scatter Width. **(B)** Heatmap showing blocks of the Top 4 genes in each defined cluster, totaling 11 distinct endocardial clusters. **(C)** Violin plots of genes associated with HSPCs (*drl, drll.2, hdr, ikzf1,* and *gata2b*) and differentiated erythrocytes (*hbae3*). **(D)** UMAP indicating the cell cycle phase of each cell. Most are in the quiescent G1 phase (4098 out of 6785 cells), with others undergoing active cell cycle in the S phase (1802 out of 6785 cells) or the G2/M phase (885 out of 6785 cells).

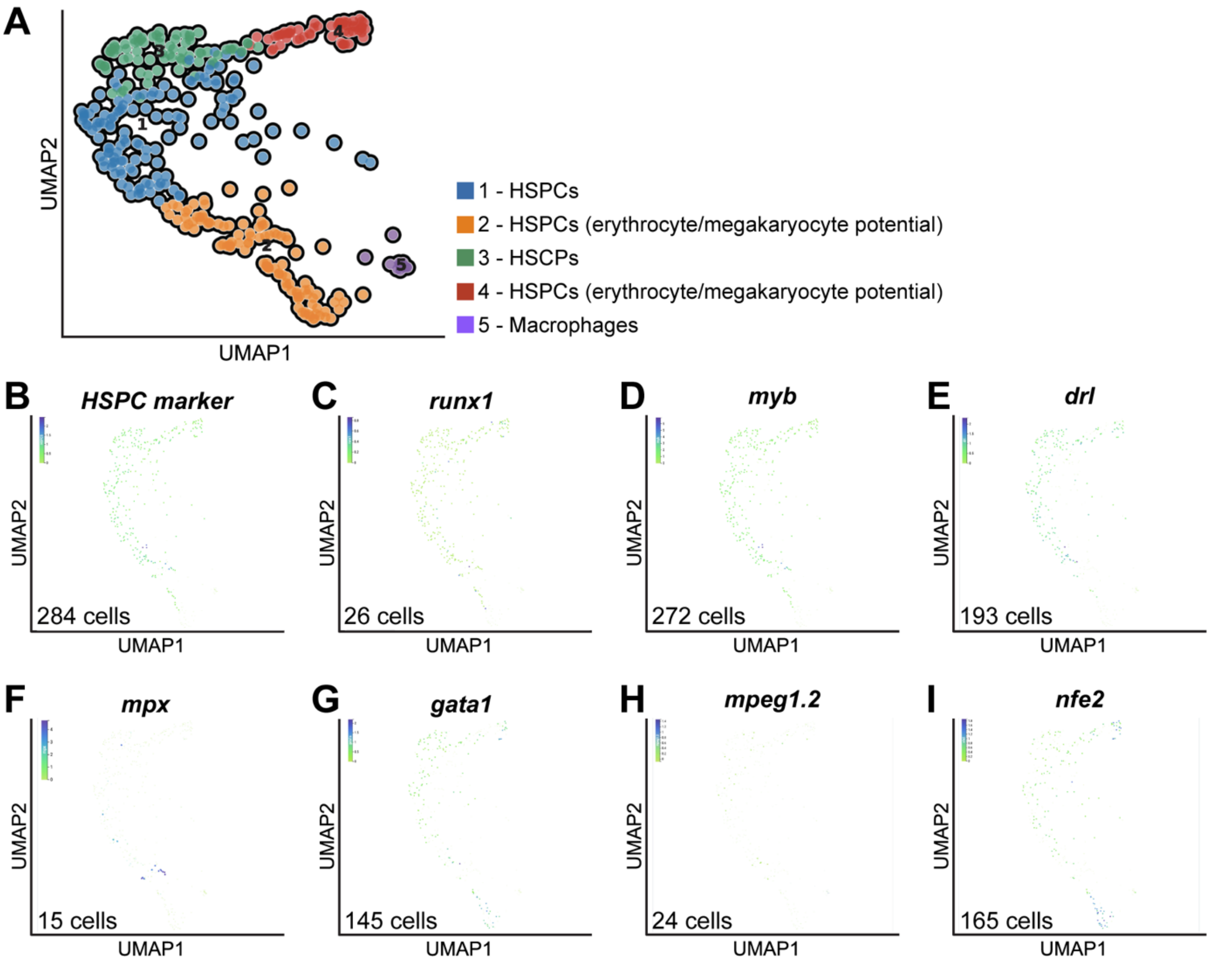

Supplemental Figure 2. Analysis of the hematopoietic endocardial cell cluster (Cluster 5) uncovers 5 sub-clusters. (A) UMAP of the endocardial hematopoietic cell cluster (Cluster 5) can be divided into 5 subclusters, including 2 HSPC clusters (Subclusters 1 and 3), 2 HPSC clusters with an erythrocyte/megakaryocyte potential (Subclusters 2 and 4), and a macrophage-like cluster (Subcluster 5). **(B-E)** UMAP of the endocardial hematopoietic cell subclusters show combined expression of key HSPC markers *myb, drl,* and *runx1* (B, 284/390 cells), as well as individual expression profiles of *runx1* (C, 26/390 cells), *myb* (D, 272/390 cells), and *drl* (E, 193/390 cells). **(F-I)** UMAP of the endocardial hematopoietic cell subcluster showing the expression of *mpx* (F, neutrophils, 15/390 cells), *gata1a* (G, erythrocytes, 145/390 cells), *mpeg1.2* (H, macrophages, 24/390 cells), and *nfe2* (I, platelets, 165/390 cells).

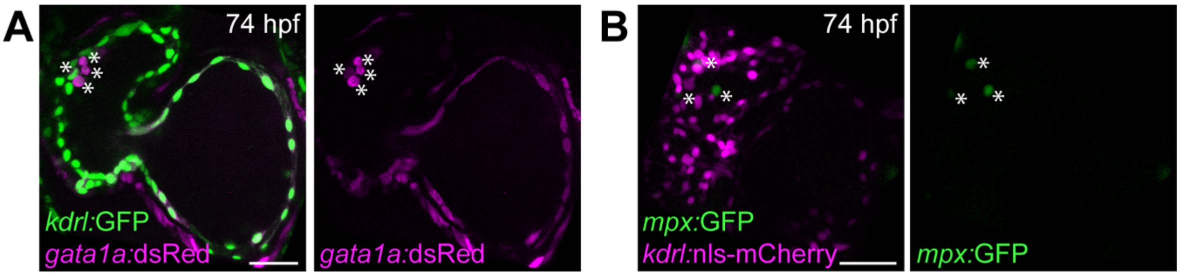

Supplemental Figure 3. Erythrocytes and neutrophils are present in the endocardial layer. Representative maximal Z projections of 74 hpf hearts with the erythrocyte-specific marker *Tg(gata1*:DsRed) (A) or the neutrophil-specific marker *Tg(mpx*:GFP) (B) expression show a population of erythrocytes and a small number of neutrophils attached to the endocardium. In all hearts examined, the hematopoietic cells appear round and undifferentiated, in contrast with mature erythrocytes and neutrophils. Scale bars = 30 µm.

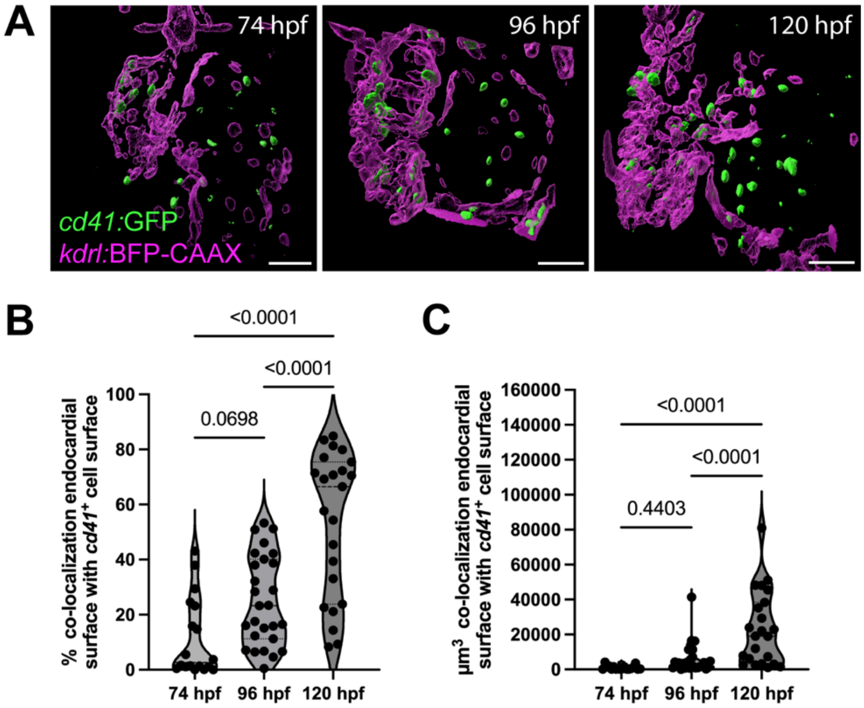

Supplemental Figure 4. Regional preferences of *cd41:*GFP^{+} cells to attach to the ventricular outer curvature area. (A) Surface rendering of *Tg(cd41*:GFP)$^{+}$*, Tg(kdrl*:BFP-CAAX)$^{+}$ hearts at 74, 96, and 120 hpf. Hematopoietic cells are noticeably present in higher numbers in the outer curvature of the ventricle. Scale bars = 30 µm. **(B, C)** Quantitative analysis of the hematopoietic and endocardial surface co-localization in the whole heart at 74, 96, and 120 hpf demonstrates that approximately 11% (856 µm^{3}), 25% (5896 µm^{3}), and 53% (23067 µm^{3}) of the cell surface is shared between the two cell populations, respectively. One-way ANOVA with Tukey's multiple comparison test was used for statistical analysis. *n*=18 (74 hpf), 27 (96 hpf), and 23 (120 hpf) hearts. Source data are provided as a Source Data file.

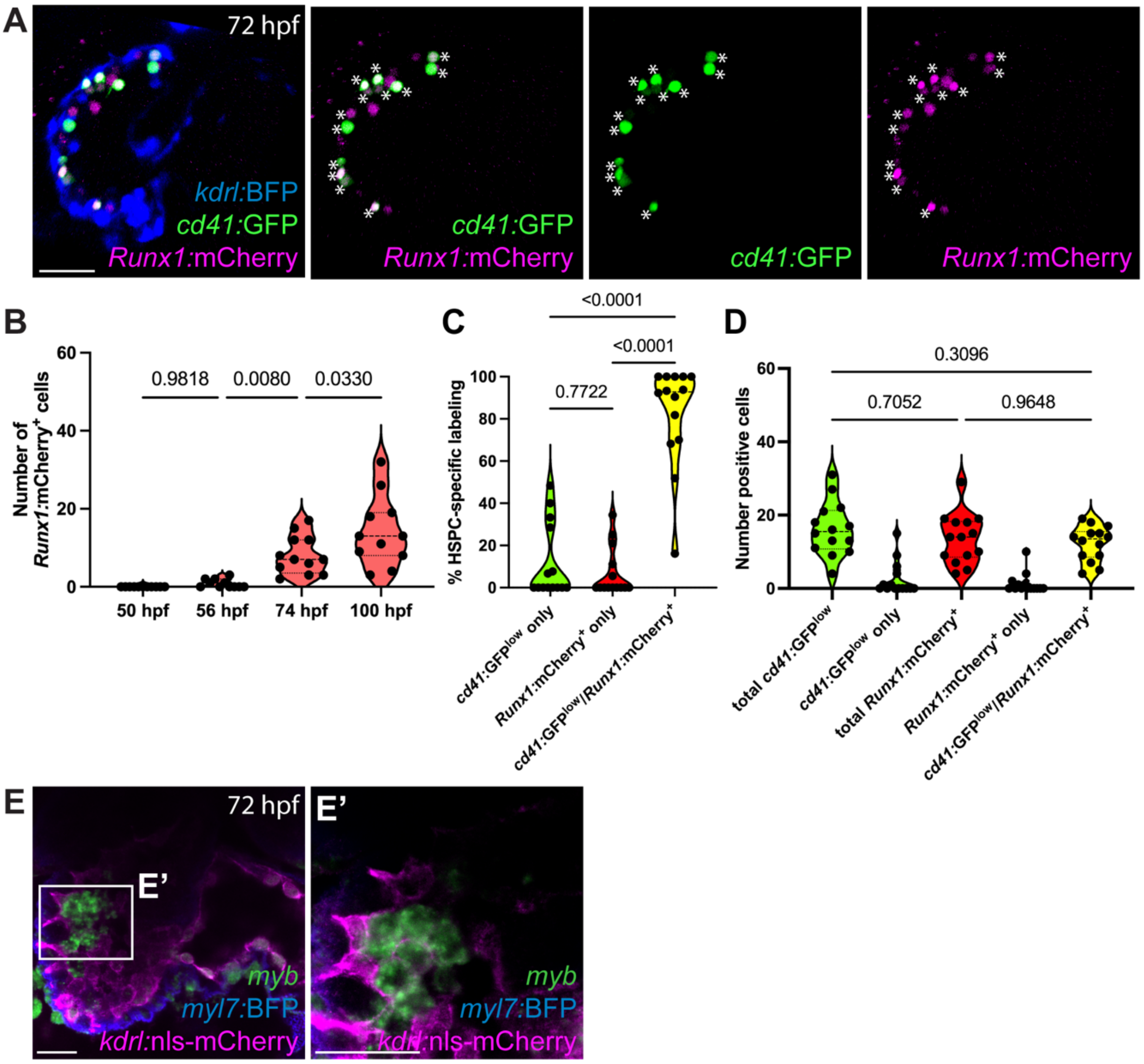
A
72 hpf
kdrl:BFP
cd41:GFP
Runx1:mCherry
B
Number of Runx1:mCherry+ cells
0.9818
0.0080
0.0330
50 hpf
56 hpf
74 hpf
100 hpf
C
<0.0001
<0.0001
0.7722
% HSPC-specific labeling
cd41:GFPlow only
Runx1:mCherry+ only
cd41:GFPlow/Runx1:mCherry+
D
0.3096
0.7052
0.9648
Number positive cells
total cd41:GFPlow
cd41:GFPlow only
total Runx1:mCherry+
Runx1:mCherry+ only
cd41:GFPlow/Runx1:mCherry+
E
72 hpf
E'
myb
myl7:BFP
kdrl:nls-mCherry

Supplemental Figure 5. HSPC markers *Tg(Runx1*:mCherry) and *myb* are expressed in the cardiac-residing hematopoietic cells. **(A)** Confocal images of *Tg(cd41:GFP), Tg(Runx1:mCherry), Tg(kdrl:BFP-CAAX)* hearts at 72 hpf reveal that most cardiac-residing HSPCs exhibit *Tg(cd41*:GFP) and *Tg(Runx1*:mCherry) co-expression (white asterisks). **(B)** Quantification of *Tg(Runx1*:mCherry)$^{+}$ cells shows progressively increased numbers of cardiac-residing HSPCs, consistent with *Tg(cd41:*GFP)$^{+}$ cells. *n*=11 (50 hpf), 11 (56 hpf), 12 (74 hpf), and 11 (100 hpf) hearts. **(C)** Almost 100% of endocardial-residing hematopoietic cells are co-labeled with both *cd41:*GFP^{low+} and *Runx1:*mCherry expression. **(D)** Quantifications of single or double-positive cells for *Tg(cd41*:GFP) and *Tg(Runx1*:mCherry) expression show no significant differences between *cd41:*GFP^{low+} or *Runx1*:mCherry expression to mark HSPCs. **(C-D)** *n*=14 hearts. **(E, E')** Fluorescence in situ hybridization shows *myb* expression in the endocardial hematopoietic cells. One-way ANOVA with Tukey's multiple comparison test was used (B-D). Scale bars = 30 µm. Source data are provided as a Source Data file.

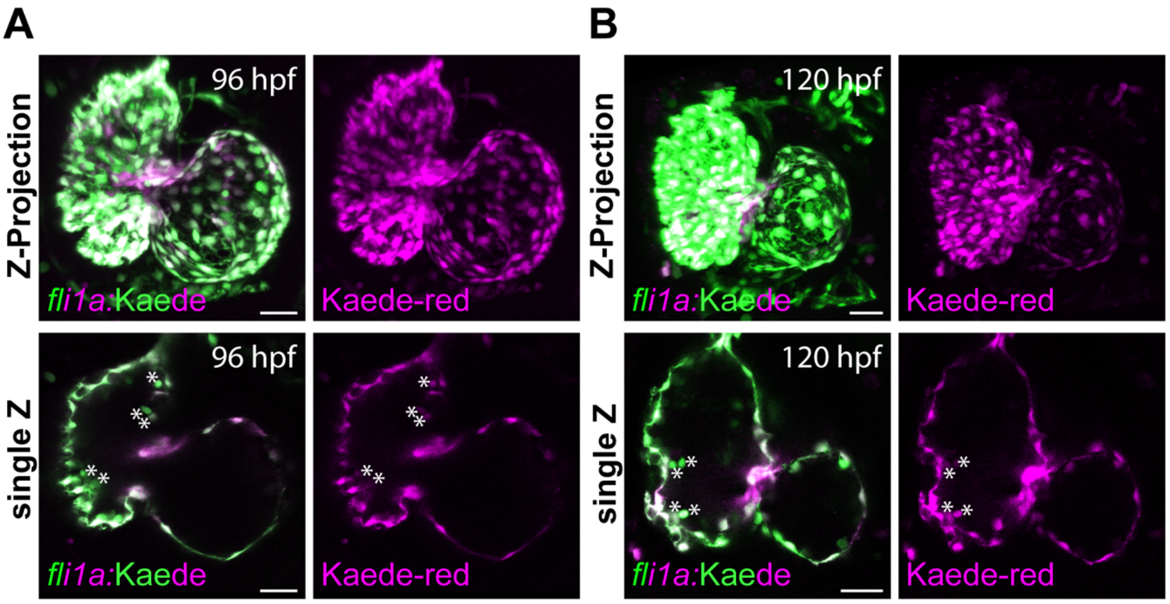

Supplemental Figure 6. Some cardiac-residing hematopoietic cells originate from and remain attached to the endocardium. (A) Representative confocal Z projection of a *Tg(fli*:Kaede)$^+$ photoconverted endocardium and a single confocal plane at 96 hpf from a larvae that had its endocardium photoconverted at 22 hpf. Kaede-red$^+$ cells were present in the luminal side of the endocardium and stayed attached during development (white asterisk). **(B)** Confocal image of 120 hpf *Tg(fli*:Kaede)$^+$ larval heart, which had its endocardium photoconverted at 22 hpf. Kaede-red$^+$ cells are observed in the luminal side of the endocardium (white asterisk). Scale bars = 30 µm.

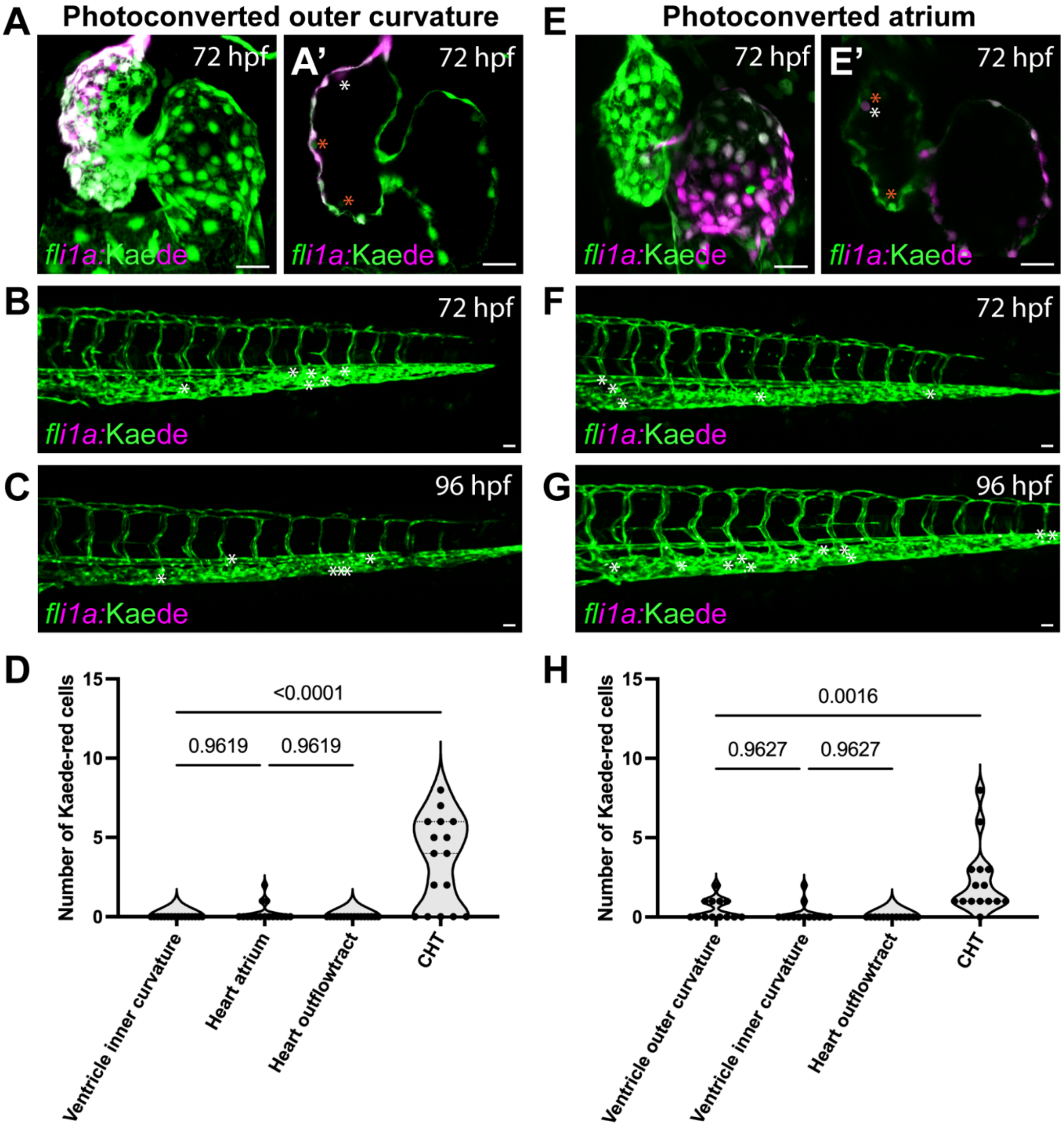
A
Photoconverted outer curvature
72 hpf
A'
72 hpf
fli1a:Kaede
fli1a:Kaede
B
72 hpf
fli1a:Kaede
C
96 hpf
fli1a:Kaede
D
Number of Kaede-red cells
15
10
5
0
<0.0001
0.9619
0.9619
Ventricle inner curvature
Heart atrium
Heart outflowtract
CHT
E
Photoconverted atrium
72 hpf
E'
72 hpf
fli1a:Kaede
fli1a:Kaede
F
72 hpf
fli1a:Kaede
G
96 hpf
fli1a:Kaede
H
Number of Kaede-red cells
15
10
5
0
0.0016
0.9627
0.9627
Ventricle outer curvature
Ventricle inner curvature
Heart outflowtract
CHT

Supplemental Figure 7. Endocardial cells from the ventricular outer curvature and the atrium contribute to cardiac-residing hematopoietic cells. (A, A') Representative confocal Z-stack projection and single plane of a 72 hpf heart, where the ventricular outer curvature EdCs had been photoconverted at 48 hpf. **(B, C)** Confocal Z-stack projection of the CHT at 72 (B) and 96 (C) hpf of larvae, which had its EdCs in the ventricular outer curvature region photoconverted. Kaede-red$^+$ cells (white asterisks) from the ventricular outer curvature endocardium are found in the CHT. **(D)** Quantification of ventricular outer curvature-derived Kaede-red$^+$ cells in other regions of the heart and the CHT at 72 hpf indicates a significant contribution to the CHT, but no contribution to other cardiac regions. *n*=16 hearts. **(E, E')** Representative confocal Z-stack projection (A) and single confocal plane of a 72 hpf heart, which had its atrial EdCs photoconverted at 48 hpf. Kaede-red$^+$ (white asterisk) and Kaede-green$^+$ (orange asterisks) cells are found on the ventricular outer curvature endocardium, indicating that atrial EdCs budded off and attached to the ventricle. **(F, G)** Confocal Z-stack projection of the CHT at 72 (F) and 96 (G) hpf of larvae with photoconverted atrial EdCs. Kaede-red$^+$ cells (white asterisks) from the atrial endocardium can be found in the CHT. **(H)** Quantification of atrial-derived Kaede-red$^+$ cells in other regions of the heart and the CHT at 72 hpf indicates mostly contribution to the CHT. *n*=15 hearts. One-way ANOVA with Tukey's multiple comparison test was used (D, H). Scale bars = 30 μm. Source data are provided as a Source Data file.

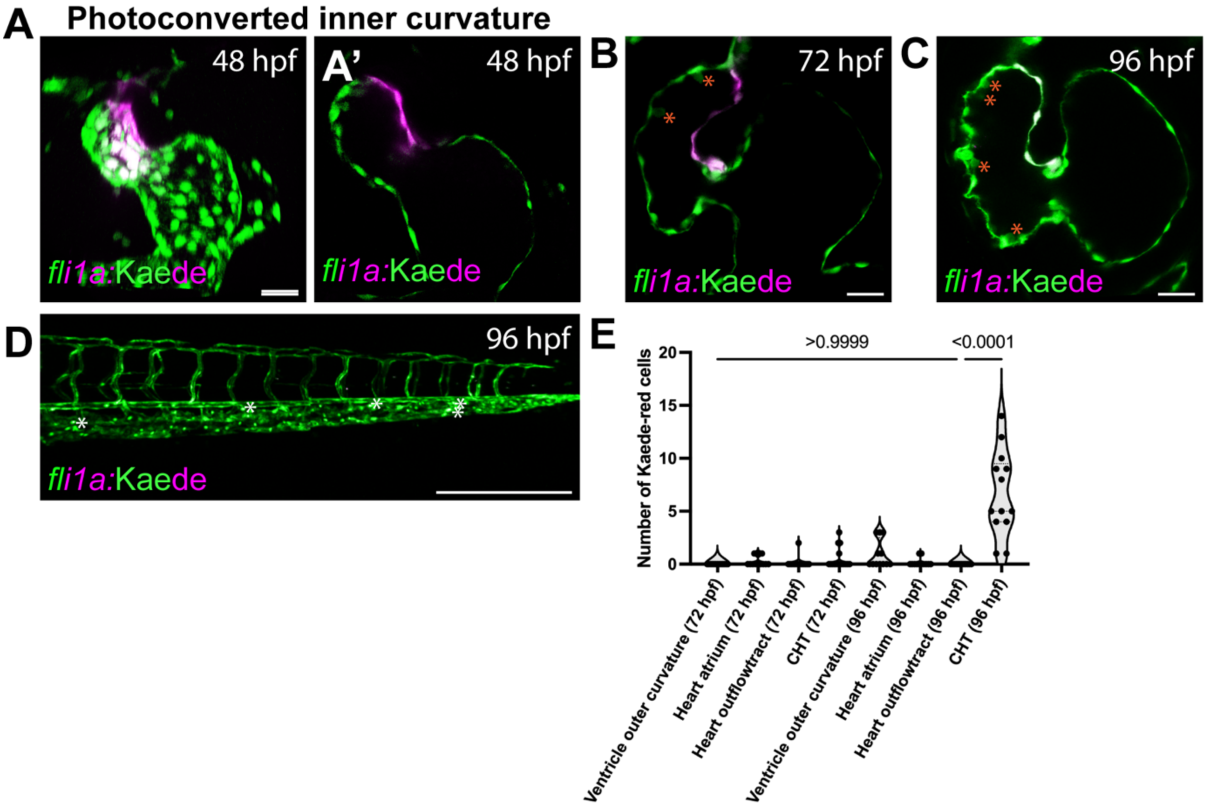

Supplemental Figure 8. The ventricular inner curvature of the endocardium only contributes a minor portion of the cardiac- or CHT-residing hematopoietic cells. (A, A') Representative confocal Z-stack projection and single plane of a 48 hpf heart, where the ventricular inner curvature EdCs had been photoconverted**. (B, C)** Single confocal plane of a *fli:*Kaede^{+} heart at 72 (B) and 96 (C) hpf, where the ventricular inner curvature endocardium had been photoconverted at 48 hpf. Only Kaede-green^{+} cells (orange asterisks) are present in the ventricular outer curvature. **(D)** Confocal Z-stack projection of the CHT at 96 hpf of larvae with photoconverted EdCs in the ventricular inner curvature. **(E)** Quantification of Kaede-red^{+} cells originating from the ventricular inner curvature of the endocardium in other regions of the heart and CHT at 72 and 96 hpf indicates only a minor contribution to hematopoietic cells present in the heart and CHT region. *n*=13, 13, 13, 16, 12, 12, 12, and 13 hearts from left to right in the violin plot graph. One-way ANOVA with Tukey's multiple comparison test was used. Scale bars = 30 µm. Source data are provided as a Source Data file.

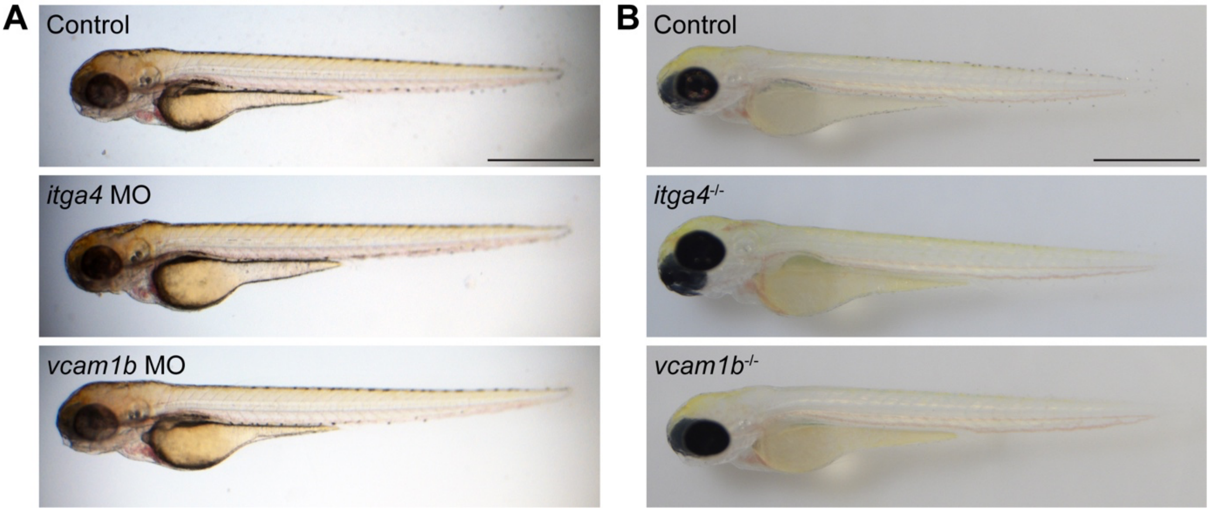

Supplemental Figure 9. *itga4* or *vcam1b* morphants and mutants do not exhibit gross morphological defects. (A, B) Whole bright-field images of *itga4* and *vcam1b* morphant (A) or mutant (B) larvae at 74 hpf do not reveal any morphological abnormalities. Scale bars = 1000 μm.

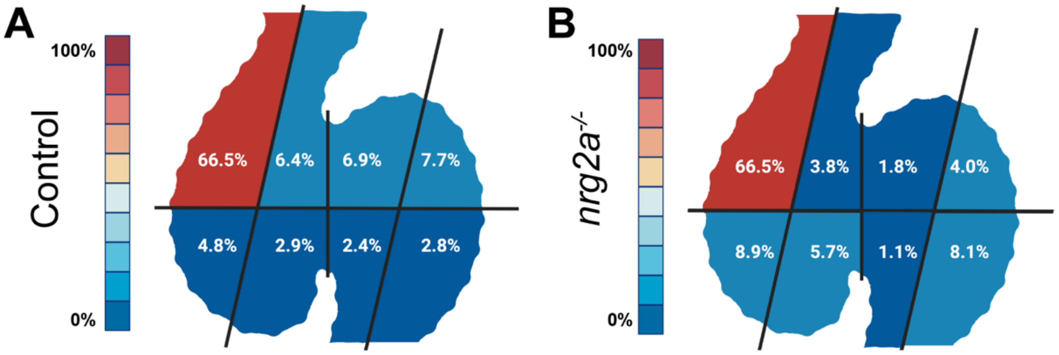

Supplemental Figure 10. Regional distribution of *cd41:*GFP$^+$ cells in *nrg2a* mutant hearts is unaltered. (A, B) No significant differences in HSPC distributions are observed between control and *nrg2a*$^{-/-}$ hearts. *n*=9 hearts. Source data are provided as a Source Data file.

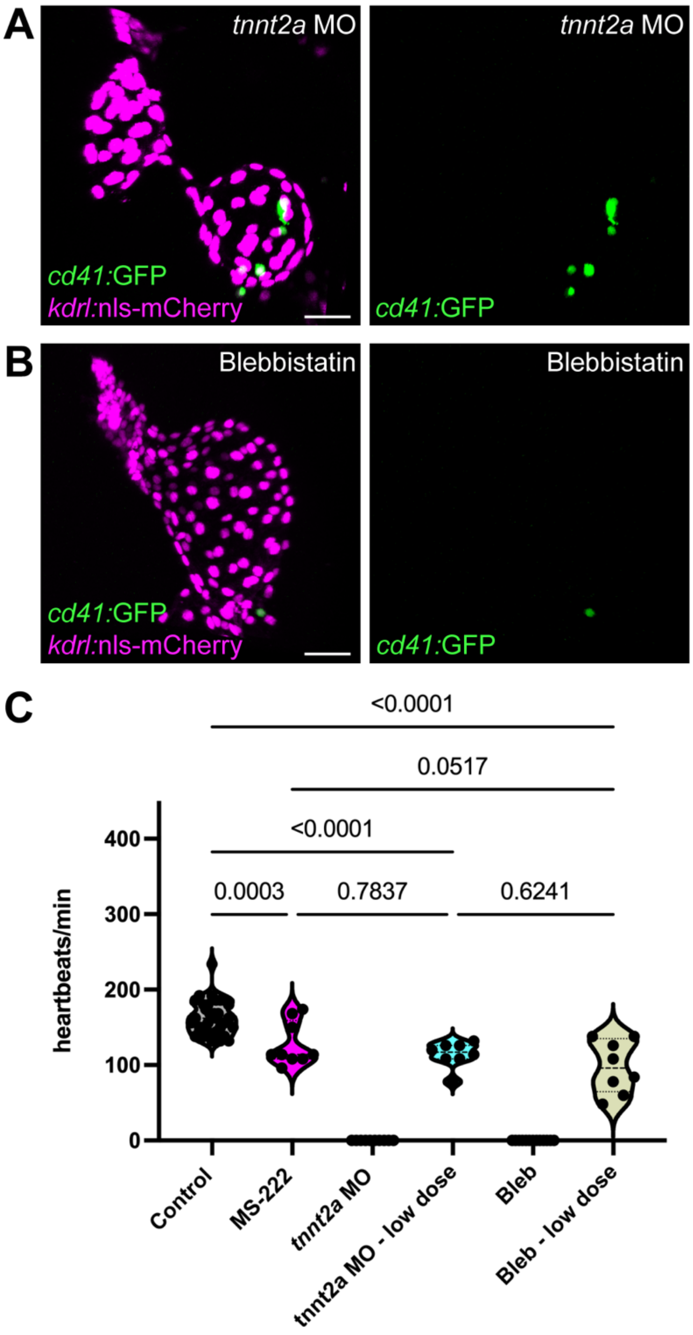
A
tnnt2a MO
tnnt2a MO
cd41:GFP
kdrl:nls-mCherry
cd41:GFP
B
Blebbistatin
Blebbistatin
cd41:GFP
kdrl:nls-mCherry
cd41:GFP
C
<0.0001
0.0517
<0.0001
0.0003
0.7837
0.6241
heartbeats/min
400
300
200
100
0
Control
MS-222
tnnt2a MO
tnnt2a MO - low dose
Bleb
Bleb - low dose

Supplemental Figure 11. Severe cardiac defects observed when contraction is completely blocked. (A, B) Hearts injected with a high concentration of *tnnt2a* morpholino **(A)** or incubated in high concentration of Blebbistatin **(B)** fail to grow and exhibit defective morphologies due to lack of cardiac contraction. No or very few *cd41:*GFP^{+} cells were found in these hearts. **(C)** Heartbeat frequency in 72 hpf control, high and low dose *tnnt2a* morpholino injections, and with 24 hour treatments of low dose MS-222/Tricaine or of high and low doses of Blebbistatin. This approach allows us to investigate reduced shear stress on endocardial hematopoietic cells while mitigating severe cardiac morphogenetic defects. *n*=7, 14, 8, 14, 33, and 36 for control (48 hpf), *gata1* morphant (48 hpf), control (54 hpf), *gata1* morphant (54 hpf), control (72 hpf), and *gata1* morphant (72 hpf) hearts, respectively. *n*=34, 10, 8, 13, 8, and 9 hearts from left to right in the violin plot graph. One-way ANOVA with Tukey's multiple comparison test was used. Scale bars = 30 µm. Source data are provided as a Source Data file.

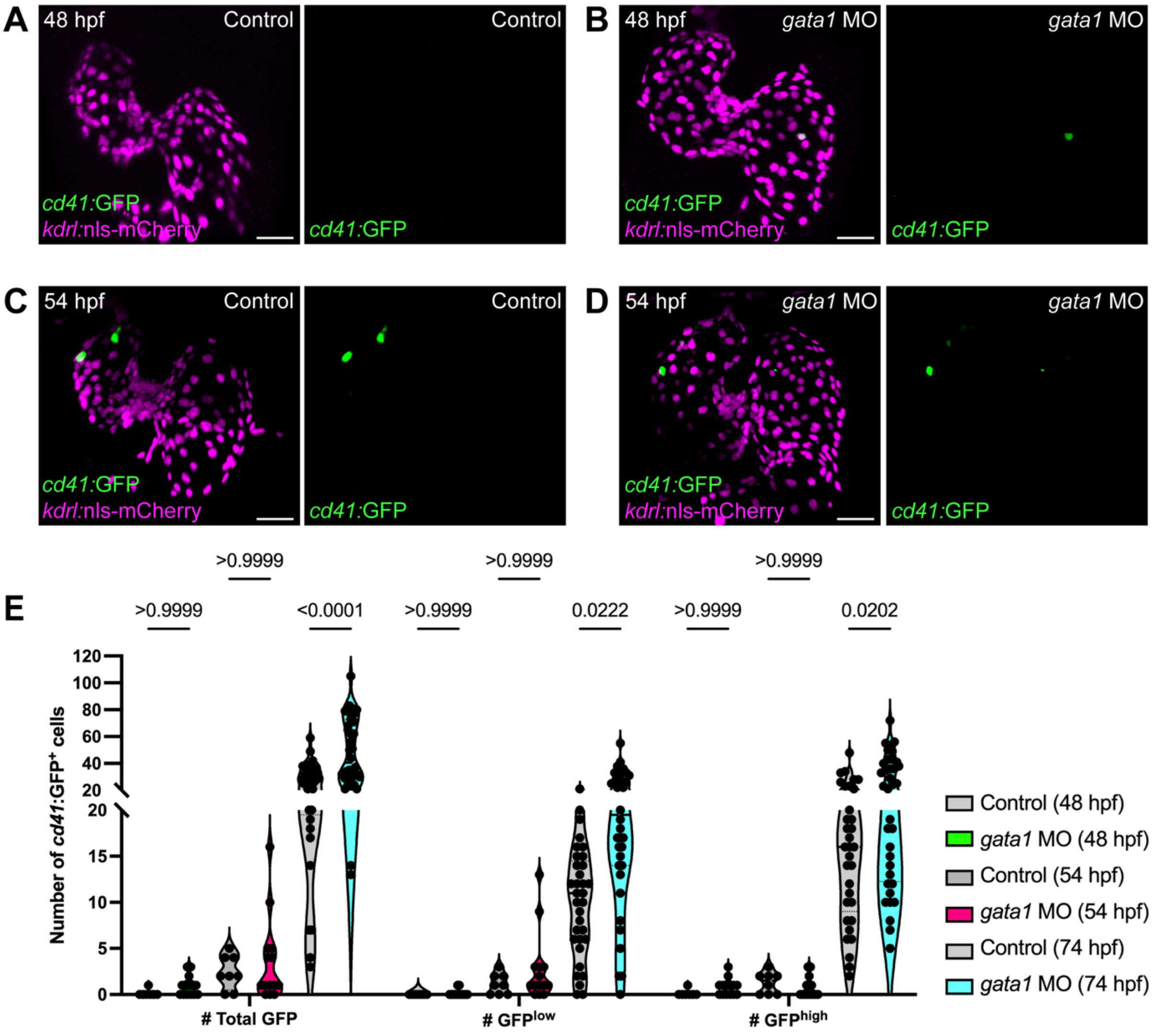

Supplemental Figure 12. Emergence of *cd41:*GFP^{+} cells in *gata1* morphants does not prematurely occur. (A-D) Representative confocal images at 48 and 54 hpf of a wild-type **(A, C)** or *gata1* morphant **(B, D)** hearts. HSPCs (GFP^{low}) and platelets (GFP^{high}) are labeled in green (*Tg(cd41:*GFP) expression) and endocardium in magenta (*Tg(kdrl:*nls-mCherry) expression). **(E)** Quantification of HSPCs and platelets at 48 and 54 hpf demonstrate no significant difference in GFP-positive cell numbers between wild-type and *gata1* morphant hearts. In contrast, at 74 hpf, there is a significant increase in HSPCs and platelets in *gata1a* morphant hearts. *n*=7, 14, 8, 14, 33, and 36 for control (48 hpf), *gata1* morphant (48 hpf), control (54 hpf), *gata1* morphant (54 hpf), control (74 hpf), and *gata1* morphant (74 hpf) hearts, respectively. Two-way ANOVA with Sidak's multiple comparison test was used for statistical analysis. Scale bars = 30 µm. Source data are provided as a Source Data file.

	CLUSTER 1 (QUIESCENT VENTRICULAR ENDOCARDIAL)	CLUSTER 2 (VALVE ENDOCARDIAL)	CLUSTER 3 (MITOTIC/DIVIDING VENTRICULAR ENDOCARDIAL)	CLUSTER 4 (VENTRICULAR ENDOCARDIAL CELL CLUSTER IN SYNTHESIS PHASE)	CLUSTER 5 (HEMATOPOIETIC ENDOCARDIAL)	CLUSTER 6 (VALVE INTERSTITIAL)	CLUSTER 7 (LYMPHATIC ENDOTHELIAL)	CLUSTER 8 (UNKNOWN CLUSTER)	CLUSTER 9 (UNKNOWN CLUSTER)	CLUSTER 10 (UNKNOWN CLUSTER)	CLUSTER 11 (UNKNOWN CLUSTER)
**1**	marcksl1b	s100a10b	hmgb2a	pcna	pfn1	sparc	gpm6ab	efemp2b	si:ch211-156j16.1	hmga1a	id1
**2**	tmem88b	crip2	hmga1a	selenoh	prdx2	col1a2	dab2	jam2a	pecam1	tubb2b	etv2
**3**	tmem88a	alcama	hmgb2b	rpa2	TXN	col5a1	marcksl1a	pecam1	myl9a	marcksl1a	id2a
**4**	marcksl1a	icn	h3f3b.1-1	nasp	COX5B	col5a2a	cpn1-1	cldn5a	gapdhs	h3f3b.1-1	jam2a
**5**	igf2b	col5a1	mki67	fen1	atp5mc1	fibinb	krt18a.1	krt8	ehd2b	etv2	cpn1-1
**6**	sdc4	CABZ01030107.1	h2az2b	cbx5	cox7a2a	serpinh1b	krt8	myl9a	cldn5b	hmgb2a	psmd3
**7**	zgc:64106	ptmaa	si:ch211-288g17.3	rpa3	cox8a	col1a1b	etv2	kdrl	jam2a	tuba8l4	her6
**8**	cnn3a	si:ch211-222l21.1	tubb4b	mcm5	cox7b	col1a1a	cldn5b	clec14a	dlc	ccna2	lmo2
**9**	ccdc187	phlda2	lbr	chaf1a	cox5aa	her6	gpr182	vwf	fbln2	si:ch211-114n24.6	sox7
**10**	CR318588.3	podxl	hmgn2	dnajc9	atp5pf	id3	rab11bb	gadd45ba	fabp3	hmgb2b	pecam1
**11**	akap12b	col5a2a	aurkb	tubb2b	atp5pd	angptl4	tgm2b	fhl1b	id2a	anp32a	sgk1
**12**	fabp11a	btbd6b	top2a	mcm6	atp5f1e	thbs1b	gas6	si:dkey-19b23.8	mcamb	plk1	rab11bb
**13**	grb10a	her15.2	tubb2b	tuba8l4	ppifb	fhl1b	kdrl	appa	acvrl1	tubb4b	si:ch211-248e11.2
**14**	CR318588.4	anxa2a	si:ch73-281n10.2	rbbp4	atp5mf	aopep	spns2	junbb	robo4	cpn1-1	si:dkey-151g10.6
**15**	45537	si:ch211-214p16.1	cks1b	mcm4	cox5ab	abi3bpb	pde4ba	BX663503.3	tmsb4x	mki67	prdx2
**16**	srgn	si:dkey-42i9.6	tuba8l4	rpa1	ldhba	f3b	si:dkey-28n18.9	adcy2b	tagln2	aurkb	dusp1
**17**	add3a	col1a2	smc2	mcm2	atp5f1b	aif1l	jun	quo	notchl	lbr	bcl6b
**18**	hapln1b	anxa1a	mad2l1	anp32b	atp5f1c	mfap2	lmo4a	ptmaa	kdrl	dab2	hey1
**19**	id2b	si:rp71-1h20.9	cdk1	sumo3b	cox7a2l	entpd1	bmp6	efnb2a	rgs5b	si:ch73-281n10.2	fabp3
**20**	ctsla	dapk1	ptmab	si:ch211-156b7.4	ndufa4l	mxra8b	jdp2b	jdp2b	cxcr4a	gpm6ab	rgs5b
**21**	rpz5	rhoab	seta	tmem88b	atp5mc3b	foxc1b	lyve1b	wnt11	etv2	lmnb2	id3
**22**	ppdpfb	calm1a	plk1	igf2b	atp5f1d	pcolcea	sox7	fosb	si:ch73-335l21.4	dek	smad7
**23**	gpr182	anxa5b	ube2c	sub1a	ahcy	cpz	calcrla	fkbp1aa	clec14a	smc2	tlx2

**24**	krt94	has2	tpx2	seta	idh2	crip2	lmo2	krt18a.1	ldb2a	cks1b	mex3b
**25**	pim1	col1a1a	ncapg	ubr7	cox6c	tnfaip6	sox4a-1	cldn5b	hopx	stmn1a	cx43
**26**	sept9a	hey2	kif23	cbx1a	uqcrq	ptmaa	zfp36l1b	LO018188.1	mylk5	gpr182	stab2
**27**	clic2	akap13	ccna2	lig1	atp5md	phlda2	pecam1	ehd2b	hey1	rab11bb	zgc:158463
**28**	insra	si:ch211-214p16.2	anp32a	hmgb2b	mibp2	smad9	rab8b	cavin1b	cxcr4b	tuba8l	fli1b
**29**	rcan1a	anxa4	spc25	rfc4	blf	hand2	nrp2b	ca16b	cfl1	top2a	gata2a
**30**	krt8	mb	ncapd2	mcm3	cox17	matn4	junbb	sik1	nova2	rab8b	fosab
**31**	palm1b	tmtc1	kif22	cbx3a	cycsb	tbx20	plk2b	krt94	tbx1	CABZ01058261.1	tcima
**32**	zfp36l1b	cd151	CABZ01058261.1	uhrf1	fabp3	fn1a	CR751602.2	FP102018.1	tie1	dut	her9
**33**	btg2	efna1b	dlgap5	marcksl1a	atp5if1b	tmem119b	nr2f1a	sox7	tmem108	si:dkey-28n18.9	cxcl18b
**34**	krt18a.1	si:ch211-1a19.3	arpp19b	cnbpa	hdr	hspa5	adcy2b	foxp4	oaz2b	kif23	plvapb
**35**	tpm4a	flt1	tuba8l	dut	atp5l	ppib	id2a	dap1b	lhx8a	kif22	gamt
**36**	mmrn2a	aldh1a2	lsm7	marcksl1b	vdac2	id2a	cyp1a	sele	ca16b	kdrl	tek
**37**	kctd12.2	tnnt2a	hmgb1b	hmgn7	atp5meb	sdc2	tie1	emid1	jam3b	pecam1	ldb2a
**38**	ramp2	grb10b	stmn1a	ranbp1	tomm7	twist1b	tspan18b	spock3	ponzr1	zgc:153867	bahcc1b
**39**	jun	arl4ab	dek	orc4	ppiab	ckap4	akap12b	edn2	hlx1	h2az2b	ppib
**40**	dusp6	grapa	cks2	rrm1	pa2g4a	ctnnb1	sesn1	atf3	jupa	ncapg	dab2
**41**	rap1b	rspo2	cenpf	snrpf	c1qbp	mmel1	UBB	ptn	krt18a.1	si:ch211-288g17.3	nrp2b
**42**	wnk1b	hbegfa	kpna2	fabp11a	sod1	si:ch211-117c9.5	pros1	tie1	zgc:153311	cdk1	rasip1
**43**	errfi1a	wasf3b	calm3a	rnaseh2a	atp5if1a	angptl7	igfbp1a	si:ch211-222l21.1	ppiab	anp32b	ybx1
**44**	dap1b	rbpms2a	cdca8	prim2	npm3	postnb	sele	limch1b	fli1a	spns2	ier2a
**45**	cnn2	eif3ea	aspm	stmn1a	cct2-1	arpc1b	CU570769.1	her9	sult2st1	akap12b	prcp
**46**	junbb	jam2a	kifc1	mcm7	nop2	angpt1	fosab	cdh5	spns2	nrp2b	jun
**47**	gpc4	aqp1a.1	cenpx	dnmt1	cct7	nanos1	si:ch211-145b13.6	s1pr5a	shroom4	mafbb	eno3
**48**	ABR	tspan7	ttk	dhfr	naa10	svild	cyp26b1	adgrl4	sema7a	actb1	atoh8
**49**	h3f3c	cd9b	cenpe	banf1	ndufab1a	cdh11	atf3	bmp16	fkbp1aa	sele	mef2cb
**50**	junba	net1	banf1	npm1a	zgc:193541	fstl1b	zgc:158343	id2a	cav1	rrm1	aldh9a1a.1

Supplemental Table 1. Top 50 most upregulated genes in each cluster of the single-cell RNA sequencing of purified endocardial cells.

**Developmental time point**	**% of hearts with *Tg(cd41:*GFP)$^+$ cells**	***Tg(cd41:*GFP)$^+$ hearts/ total hearts**
50 hpf	12%	2/17
56 hpf	52%	11/21
74 hpf	96%	102/106
98 hpf	100%	43/43
120 hpf	100%	34/34
7 dpf	100%	33/33
10 dpf	100%	19/19

Supplemental Table 2. Percentage of hearts containing *Tg(cd41:*GFP)$^+$ cells at the indicated developmental time point.
