# Supplementary material for: The heart is a resident tissue for hematopoietic stem and progenitor cells in zebrafish
Source: Nat Commun. 2024 Aug 31;15:7589. doi: 10.1038/s41467-024-51920-7 (PMC11366026; doi:10.1038/s41467-024-51920-7)
Supplement: Supplementary file 3 — Description of Additional Supplementary Files [file 41467_2024_51920_MOESM3_ESM.pdf]

Description of Additional Supplementary Files

File Name: Supplementary Movie 1
Description: **Visualization of beating *Tg(cd41*:GFP)$^+$, *Tg(kdrl*:nls-mCherry)$^+$ heart reveals endocardial attachment of HSPCs.** Dynamic representation of a zebrafish heart displaying rhythmic contractions in a single z-slice. The heartbeat is captured in real-time, showcasing the cardiac activity. *cd41:*GFP-labeled HSPCs (green) are stably attached to the endocardial cells in the ventricle (left side of video).

File Name: Supplementary Movie 2
Description: **Visualization of beating *Tg(kdrl:*GFP)$^+$, *Tg(gata1:*DsRed)$^+$ heart reveals endocardial attachment of erythrocyte progenitors.** Dynamic representation of a zebrafish heart displaying rhythmic contractions in a single z-slice. *gata1:*DsRed-labeled erythrocytes show circulating mature erythrocytes in the heart, and rounded, immature erythrocyte progenitors attached to the endocardial cells in the ventricle (right side of video).

File Name: Supplementary Movie 3
Description: **3D view of *Tg(cd41*:GFP)$^+$, *Tg(kdrl*:nls-mCherry)$^+$ heart, with *cd41*:GFP-labeled HSPCs attached to the inner lining of the endocardium.** Z-stack and -plane movie through the surface rendering of *cd41* and *kdrl* expression in a 72 hpf heart highlighting the location and shared surface areas of the two cell populations.

File Name: Supplementary Movie 4
Description: **Light sheet high-speed heartbeat recording of a *Tg(cd41*:GFP)$^+$, *Tg(kdrl*:nls-mCherry)$^+$ heart shows the activation of *cd41:*GFP expression.** A single section plane focusing on a representative endocardial cell within the inner curvature of the ventricle. Initially, at the start of imaging (0 minutes), no expression of *cd41*:GFP is observed. At 30 minutes, *cd41* expression begins to appear, and by 60 minutes, strong expression of *cd41*:GFP is evident, marked by a yellow asterisk indicating the specific location within the inner curvature of the ventricle.

File Name: Supplementary Movie 5
Description: **Brightfield recording of a zebrafish heartbeat in control embryos.** The image was captured using a 20x objective lens, illustrating the rhythmic contractions of the zebrafish heart.

File Name: Supplementary Movie 6
Description: **Brightfield recording of a zebrafish heartbeat in *itga4*$^{-/-}$ embryos.** The heartbeat shows no differences comparing control and *itga4*$^{-/-}$ mutant embryos.

File Name: Supplementary Movie 7
Description: **Brightfield recording of a zebrafish heartbeat in *vcam1b*$^{-/-}$ embryos.** No heartbeat alterations are visible compared to control and *vcam1b*$^{-/-}$ mutant embryos.

File Name: Supplementary Movie 8
Description: **Brightfield recording of a zebrafish heartbeat in *vcam1b-/-* embryos.** No heartbeat alterations are visible compared to control and *vcam1b-/-* mutant embryos.
