# Supplementary material for: The heart is a resident tissue for hematopoietic stem and progenitor cells in zebrafish
Source: Nat Commun. 2024 Aug 31;15:7589. doi: 10.1038/s41467-024-51920-7 (PMC11366026; doi:10.1038/s41467-024-51920-7)
Supplement: Supplementary file 12 — Reporting Summary [file 41467_2024_51920_MOESM12_ESM.pdf]

nature portfolio

Corresponding author(s): Felix Gunawan

Last updated by author(s): Aug 5, 2024

Reporting Summary

Nature Portfolio wishes to improve the reproducibility of the work that we publish. This form provides structure for consistency and transparency in reporting. For further information on Nature Portfolio policies, see our Editorial Policies and the Editorial Policy Checklist.

Statistics

For all statistical analyses, confirm that the following items are present in the figure legend, table legend, main text, or Methods section.

n/a	Confirmed	
☐	☒	The exact sample size (n) for each experimental group/condition, given as a discrete number and unit of measurement
☐	☒	A statement on whether measurements were taken from distinct samples or whether the same sample was measured repeatedly
☐	☒	The statistical test(s) used AND whether they are one- or two-sided<br>*Only common tests should be described solely by name; describe more complex techniques in the Methods section.*
☒	☐	A description of all covariates tested
☐	☒	A description of any assumptions or corrections, such as tests of normality and adjustment for multiple comparisons
☐	☒	A full description of the statistical parameters including central tendency (e.g. means) or other basic estimates (e.g. regression coefficient) AND variation (e.g. standard deviation) or associated estimates of uncertainty (e.g. confidence intervals)
☐	☒	For null hypothesis testing, the test statistic (e.g. F, t, r) with confidence intervals, effect sizes, degrees of freedom and P value noted<br>*Give P values as exact values whenever suitable.*
☒	☐	For Bayesian analysis, information on the choice of priors and Markov chain Monte Carlo settings
☒	☐	For hierarchical and complex designs, identification of the appropriate level for tests and full reporting of outcomes
☒	☐	Estimates of effect sizes (e.g. Cohen's d, Pearson's r), indicating how they were calculated

Our web collection on statistics for biologists contains articles on many of the points above.

Software and code

Policy information about availability of computer code

Data collection	Zen 2.3 SP1 FP3 black, Zen 2011 SP3 black, Zen 2.6 Blue were used for imaging.
Data analysis	Bitplane Imaris Version 9.9.1 and 10.01 (UK), GraphPad PRISM Version 10 (USA), ImageJ (Fiji package set; NIH, USA)

For manuscripts utilizing custom algorithms or software that are central to the research but not yet described in published literature, software must be made available to editors and reviewers. We strongly encourage code deposition in a community repository (e.g. GitHub). See the Nature Portfolio guidelines for submitting code & software for further information.

Data

Policy information about availability of data

All manuscripts must include a data availability statement. This statement should provide the following information, where applicable:

- Accession codes, unique identifiers, or web links for publicly available datasets
- A description of any restrictions on data availability
- For clinical datasets or third party data, please ensure that the statement adheres to our policy

The authors declare that the data supporting the findings of this study are available within the paper and its supplementary information files. Source data are provided with this paper. The single cell sequencing dataset is available in the Gene Expression Omnibus database under accession number GSE269378, which is now already public.

Research involving human participants, their data, or biological material

Policy information about studies with human participants or human data. See also policy information about sex, gender (identity/presentation), and sexual orientation and race, ethnicity and racism.

Reporting on sex and gender	*Use the terms sex (biological attribute) and gender (shaped by social and cultural circumstances) carefully in order to avoid confusing both terms. Indicate if findings apply to only one sex or gender; describe whether sex and gender were considered in study design; whether sex and/or gender was determined based on self-reporting or assigned and methods used. Provide in the source data disaggregated sex and gender data, where this information has been collected, and if consent has been obtained for sharing of individual-level data; provide overall numbers in this Reporting Summary. Please state if this information has not been collected. Report sex- and gender-based analyses where performed, justify reasons for lack of sex- and gender-based analysis.*
Reporting on race, ethnicity, or other socially relevant groupings	*Please specify the socially constructed or socially relevant categorization variable(s) used in your manuscript and explain why they were used. Please note that such variables should not be used as proxies for other socially constructed/relevant variables (for example, race or ethnicity should not be used as a proxy for socioeconomic status). Provide clear definitions of the relevant terms used, how they were provided (by the participants/respondents, the researchers, or third parties), and the method(s) used to classify people into the different categories (e.g. self-report, census or administrative data, social media data, etc.) Please provide details about how you controlled for confounding variables in your analyses.*
Population characteristics	*Describe the covariate-relevant population characteristics of the human research participants (e.g. age, genotypic information, past and current diagnosis and treatment categories). If you filled out the behavioural & social sciences study design questions and have nothing to add here, write "See above."*
Recruitment	*Describe how participants were recruited. Outline any potential self-selection bias or other biases that may be present and how these are likely to impact results.*
Ethics oversight	*Identify the organization(s) that approved the study protocol.*

Note that full information on the approval of the study protocol must also be provided in the manuscript.

Field-specific reporting

Please select the one below that is the best fit for your research. If you are not sure, read the appropriate sections before making your selection.

☒ Life sciences ☐ Behavioural & social sciences ☐ Ecological, evolutionary & environmental sciences

For a reference copy of the document with all sections, see nature.com/documents/nr-reporting-summary-flat.pdf

Life sciences study design

All studies must disclose on these points even when the disclosure is negative.

Sample size	To ensure the reproducibility and statistical power, all experiments were performed in at least 3 biological replicates with minimum sample sizes of 8 embryos/larvae.
Data exclusions	No data were excluded from the study.
Replication	A minimum of 3 biological replicates were performed for each experiment to ensure reproducibility of our experiments.
Randomization	Randomization was achieved in this study due to random selection of transgenic and/or mutant embryos for cardiac analysis. Hence, group assignment could not be predicted and no additional kinds of randomization were needed, since all experiments were repeated at least 3 times on different days.
Blinding	A blinding of experiments was not possible due to the genetic validation of zebrafish strains and obvious phenotypes of pharmaceutical treatments on cardiac morphology or blood flow. The potential for observer bias of our data sets was minimized by repetition of experiments, imaging and quantitative analysis by multiple authors (Dorothee Bornhorst, Amulya Hejjaji, Lena Steuter, Nicole Woodhouse, Felix Gunawan).

Reporting for specific materials, systems and methods

We require information from authors about some types of materials, experimental systems and methods used in many studies. Here, indicate whether each material, system or method listed is relevant to your study. If you are not sure if a list item applies to your research, read the appropriate section before selecting a response.

Materials & experimental systems

n/a	Involved in the study	
☐	☒	Antibodies
☒	☐	Eukaryotic cell lines
☒	☐	Palaeontology and archaeology
☐	☒	Animals and other organisms
☒	☐	Clinical data
☒	☐	Dual use research of concern
☒	☐	Plants

Methods

n/a	Involved in the study	
☒	☐	ChIP-seq
☐	☒	Flow cytometry
☒	☐	MRI-based neuroimaging

Antibodies

Antibodies used	Anti-eGFP antibody (Thermo Fisher A-11122, polyclonal rabbit) and Alexa Fluor 488 (Thermo Fisher A32723, goat secondary antibody) were used in the study.
Validation	The antibody has been validated by the supplier and our previous publication:<br><br>Gunawan, F. et al. Focal adhesions are essential to drive zebrafish heart valve morphogenesis. J Cell Biol 218, 1039-1054, doi:10.1083/jcb.201807175 (2019).

Animals and other research organisms

Policy information about studies involving animals; ARRIVE guidelines recommended for reporting animal research, and Sex and Gender in Research

Laboratory animals	Zebrafish (Danio rerio), strain: Tüb/AB, adult fish (both males and females) were used in the study. Experiments were performed up until 120 hpf. 7 and 10 dpf larva were first fixed in paraformaldehyde prior to imaging. Specific lines (transgenic lines and mutants) are listed in the Methods section of the paper.
Wild animals	*Provide details on animals observed in or captured in the field; report species and age where possible. Describe how animals were caught and transported and what happened to captive animals after the study (if killed, explain why and describe method; if released, say where and when) OR state that the study did not involve wild animals.*
Reporting on sex	Sex was not a factor in this study.
Field-collected samples	*For laboratory work with field-collected samples, describe all relevant parameters such as housing, maintenance, temperature, photoperiod and end-of-experiment protocol OR state that the study did not involve samples collected from the field.*
Ethics oversight	Zebrafish were handled at the University of Münster, where the ethical guidelines outlined by the state of North Rhine-Westphalia were adhered to and the handling was conducted under the supervision of the veterinary authorities of the city of Münster, at the Max Planck Institute for Heart and Lung Research as approved by the Animal Protection Committee of the Regierungspräsidium Darmstadt, or at the University of Wisconsin-Madison in accordance with their Institutional Animal Care and Use Committee guidelines.

Note that full information on the approval of the study protocol must also be provided in the manuscript.

Plants

Seed stocks	*Report on the source of all seed stocks or other plant material used. If applicable, state the seed stock centre and catalogue number. If plant specimens were collected from the field, describe the collection location, date and sampling procedures.*
Novel plant genotypes	*Describe the methods by which all novel plant genotypes were produced. This includes those generated by transgenic approaches, gene editing, chemical/radiation-based mutagenesis and hybridization. For transgenic lines, describe the transformation method, the number of independent lines analyzed and the generation upon which experiments were performed. For gene-edited lines, describe the editor used, the endogenous sequence targeted for editing, the targeting guide RNA sequence (if applicable) and how the editor was applied.*
Authentication	*Describe any authentication procedures for each seed stock used or novel genotype generated. Describe any experiments used to assess the effect of a mutation and, where applicable, how potential secondary effects (e.g. second site T-DNA insertions, mosiacism, off-target gene editing) were examined.*

Flow Cytometry

Plots

Confirm that:

☒ The axis labels state the marker and fluorochrome used (e.g. CD4-FITC).

☒ The axis scales are clearly visible. Include numbers along axes only for bottom left plot of group (a 'group' is an analysis of identical markers).

☒ All plots are contour plots with outliers or pseudocolor plots.

☒ A numerical value for number of cells or percentage (with statistics) is provided.

Methodology

Sample preparation	Hearts from 48- and 72-hpf Tg(kdrl:nls-mCherry); Tg(cd41:GFP) larvae were manually dissected in DMEM + 10% FBS. Hearts were centrifuged for 60 seconds at 3000 rpm, washed with 1 mL Hanks' Balanced Salt Solution and dissociated into single cells by incubating in 100 µl Enzyme 1 and 5 µl Enzyme 2 (Pierce Cardiomyocyte Dissociation Kit, Thermo Fisher Scientific, Cat#88281) for 20 minutes at 300 rpm in a 30°C shaker. The samples were centrifuged for 5 minutes at 3000 rpm, the supernatant was discarded, and fresh medium was added to the dissociated cells and passed through 40 µM-filter polystyrene 5ml tubes.
Instrument	Cells were sorted using the BD FACSAria™ III (BD Biosciences) instrument.
Software	The software used for sorting and analysis is BD FACSDiva v8.0.1
Cell population abundance	35.5% were positive for only the mCherry marker (P4), and 5.6% cells were positive for both GFP and mCherry markers (P5). The population hierarchy to visualize the gating strategy is included in Figure Supplement 1A.
Gating strategy	Out of 26,419 events, 12,426 events were gated as cells (P1). After doublet exclusion with FSC-H vs FSC-A parameters (P2), the remainder 98.6% events (12,250 cells) were gated for live cells (P3) 67.5% (8,270 cells), using DAPI as a marker for dead cells. From the live cells, 35.5% (2,935 cells) were positive for the mCherry marker (P4), and 5.6% (464) cells were positive for both GFP and mCherry markers (P5).<br>Negative controls of non-fluorescent hearts or single-color fluorescent hearts were prepared to define the sorting gates. The population hierarchy to visualize the gating strategy is included in Figure Supplement 1A.

☒ Tick this box to confirm that a figure exemplifying the gating strategy is provided in the Supplementary Information.
